# Surveillance, Epidemiology, and End Results database and propensity score matching analysis of postoperative radiotherapy for non‐malignant meningioma: A retrospective cohort study

**DOI:** 10.1002/cam4.6177

**Published:** 2023-05-31

**Authors:** Yong’an Jiang, Peng Chen, JiaWei Liang, JiaHong Cai, Yi Zhang, HengYi Fan, RaoRao Yuan, Wenxin Zheng, ShiQi Cheng, Yan Zhang

**Affiliations:** ^1^ Department of Neurosurgery The Second Affiliated Hospital of Nanchang University Nanchang P. R. China; ^2^ Nanchang University Nanchang P. R. China

**Keywords:** Kaplan–Meier (KM) analysis, non‐malignant meningiomas, postoperative radiotherapy, propensity score matching (PSM), Surveillance, Epidemiology, and End Results (SEER)

## Abstract

**Background:**

The clinical effect of postoperative radiotherapy (PORT) in non‐malignant meningioma (NMM) has not been well explored.

**Methods:**

A total of 8629 patients with NMM (surgery alone group: *n* = 7716, postoperative radiotherapy group: *n* = 913) were obtained from the Surveillance, Epidemiology, and End Results database. Patient profiles were matched by 1:1 propensity score matching (PSM). Logistic regression analysis was performed to identify factors associated with PORT versus surgery alone (SA). Univariate and multivariate Cox regression analyses determined prognostic variables with overall survival (OS) in NMM. Subgroup analyses were performed with Cox proportional hazards regression models.

**Results:**

All the SA (*n* = 7716) and PORT (*n* = 913) groups were included. Women with PORT (66.3%) and SA (70.9%) were almost twice as likely as men, and tumors with benign behaviors in the SA group were almost seven times more frequent than those with malignant characteristics. We explored the demographic, clinical characteristics, and prognostic factors in NMM. Laterality, surgery, tumor size, diagnosis year, age, and tumor behavior were associated with PORT versus SA. Patients treated with PORT had better OS than those treated with SA (*p* = 0.03). After PSM, PORT remained comparable to SA (hazard ratio 0.56, 95% confidence interval 0.35–0.88, *p* = 0.013). In the subgroup analysis of PORT treatment, borderline malignant behavior increased the death risk by 23%, while other variables did not have a significant clinical benefit (*p* > 0.05).

**Conclusions:**

Borderline malignant behavior should be considered seriously, and the PORT regimen should be actively implemented for patients with benign meningiomas.

## 1 INTRODUCTION

Meningiomas are primary intracranial tumors, with majority of them being benign. Non‐malignant meningiomas (NMMs) are the most common histological type, accounting for more than half of the primary brain and central nervous systems.^1^ Following the enactment of the Benign Brain Tumor Cancer Registries Amendment Act, there has been increased research on NMMs.^2^ NMMs cause a series of damage by compressing brain tissues in various anatomical regions, which vary patient‐to‐patient. In 2007, according to the WHO histological classification of meningiomas, 80%–90% were benign Grade I tumors, 5%–15% were atypical or borderline malignant Grade II tumors, and 1%–3% were malignant Grade III tumors, and for each grade, postoperative recurrence was found in 7%–20%, 30%–40%, and 50%–80%, respectively.^3^, ^4^ Cao et al.^5^ analyzed the Surveillance, Epidemiology, and End Results (SEER) database to identify that benign and borderline meningiomas in Black individuals have poor prognosis and that surgery may improve survival.

Postoperative radiotherapy (PORT) has been shown to improve the prognosis of WHO Grade III meningiomas, and conventional PORT has become the standard treatment.^6^, ^7^ The application of PORT in NMMs is currently controversial. For WHO Grade I, the National Comprehensive Cancer Network and EZNO guidelines recommend PORT in incompletely resected tumors or symptomatic patients. Although PORT is unsuitable for patients with completely resected WHO Grade I meningioma, radiation therapy (with or without surgery) can be considered for those with WHO Grade II meningioma.^8^, ^9^ A recent study reported that atypical meningiomas (non‐malignant, classified according to the CBTRUS report ICD‐O‐3 code^10^) receiving PORT had worse survival outcomes or no survival difference with PORT.^11^, ^12^

We performed a large population‐based study to determine the effect of PORT on NMMs using propensity score matching (PSM) to eliminate baseline differences between samples. We also compared the overall survival (OS) and the impact of clinical subgroup on survival. To the best of our knowledge, this study is the first to report treatment differences in PORT in a PSM‐matched patient population.

## 2 MATERIALS AND METHODS

### 2.1 Study data collection

**FIGURE 1:**
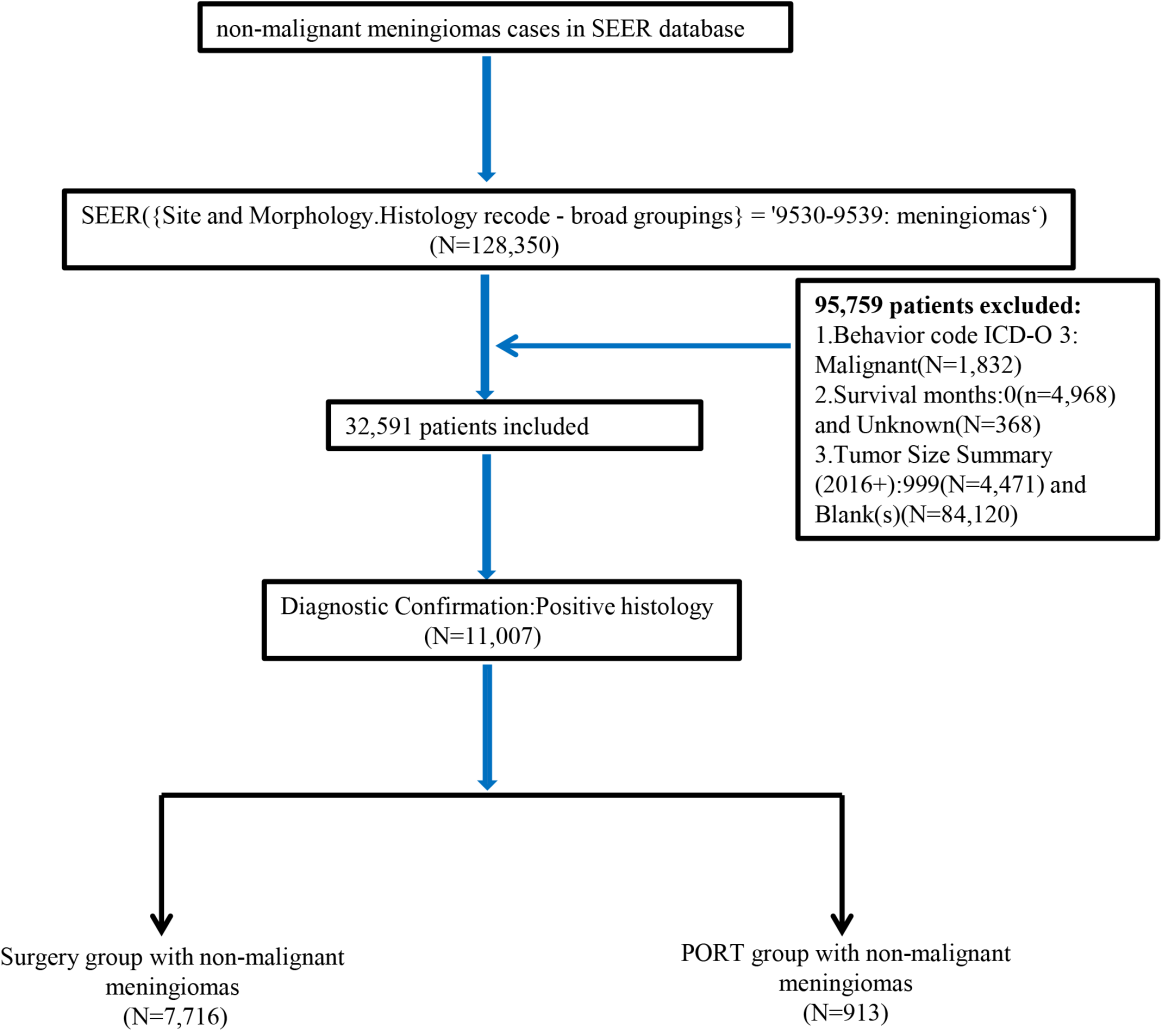
Study flowchart.

Patients included in the study were selected from all 17 registries (2000–2019) in the Online Access website, SEER database. We selected data from 2016 to 2019. The inclusion criteria included patients aged ≥20 years with histologically verified NMMs (histology recode, broadening groupings: 9530–9539 meningiomas). The study design is shown in Figure 1. The study was exempted from the institutional ethical review board approval process. The work has been reported in line with the STROCSS criteria.^13^

### 2.2 Selection of variables

In patients with histologically positive NMMs, data for the following variables were collected: sex (female, male), laterality (left, right, other), surgery (subtotal resection [STR]), gross total resection (GTR), marital status (married, separated, other), tumor size at diagnosis (≥42 mm, <42 mm, optimal size is defined by the “survminer” package, version:0.4.9), race (Black, White, other/unknown), age (20–39, 40–59, 60–79, and 80+ years), diagnosis year (2016, 2017, 2018, and 2019), behavior code (benign, borderline). Any other treatment and patients with incomplete surgical records were excluded from the surgical cohort. For the PORT cohort, data on preoperative radiotherapy, chemotherapy, and other treatments were excluded. Survival data included survival status (dead and alive) and survival month (excluded survival time is 0). The primary outcome was OS in patients with NMM receiving PORT.

### 2.3 Statistical analysis

All patients were grouped according to the treatment modality: SA and PORT. The patients' clinical characteristics were described as categorical variables (%), and distributions between the groups were compared using the Mann–Whitney *U* test, chi‐square analysis, and Fisher's exact test (where applicable). Univariate and multivariate logistic regression analyses were used to investigate the demographics associations and clinical characteristics for the relationship between PORT and SA in NMM. Prognostic characteristics were assessed for univariate Cox (*p* < 0.05) and multivariate Cox (*p* < 0.01) regression models. The Kaplan–Meier (KM) method was used to assess survival in the SA and PORT groups and clinically characterized subgroups. The risk was expressed as a hazard ratio (HR) with a 95% confidence interval (CI) to investigate the effect on survival outcomes between subgroups. All statistical analysis results in this study were generated in the R environment (R version 4.1.3, https://www.r‐project.org). A *p*‐value < 0.05 was considered statistically significant.

### 2.4 PSM

To simulate a randomized trial, 1:1 PSM was used to reduce selection bias, and the R package “MatchIt, version:4.5.0” performed “nearest” matching of baseline data of patients in the SA and PORT groups,^14^, ^15^ with caliper set to 0.02. Classification variables were compared using the chi‐square test. The variables considered in the PSM analysis included sex, laterality, surgery, marital status, tumor size, race, age, diagnosis year, and behavior code. After PSM, the KM method was used to estimate OS and draw the survival curves. Our study used the log‐rank test to identify OS‐related prognostic variables in different patient groups. Cox proportional hazard regression models were used to assess HR for OS using 95% CI.

## 3 RESULTS

### 3.1 Clinical characteristics of patients with NMM

This study included 8629 patients with NMM (Table 1), divided into SA (*n* = 7716) and PORT groups (*n* = 913). The significant clinical differences between the two groups are shown in Table 2. Compared with the SA group, the PORT group had more women (66.3% vs. 70.9%), left side tumor (45.9% vs. 38.5%), right side tumor (40.9% vs. 38.9%), and patients with STR (53.5% vs. 33.0%), fewer patients with GTR (46.5% vs. 67.0%), and similar married status (58.5% vs. 58.0%). Moreover, compared with the SA group, the PORT group consisted of more number of patients with tumors ≥42 mm (58.5% vs. 39.6%). Race was comparable in both the groups (11.1% vs. 11.3%, Black; 73.5% vs. 76.0%, White). In terms of patient age, compared to the PORT group, the SA group consisted of more number of patients aged 20–39 and 40–59 years (11.7% vs. 9.3%, 45.2% vs. 39.9%) and fewer number of patients aged 60–79, and 80+ years (41.3% vs. 45.3%, 1.8% vs. 5.4%). The proportion of patients in the PORT group was similar for each year of diagnosis (28.0%, 23.2%, 25.0%, and 23.8% in 2016, 2017, 2018, and 2019, respectively). The PORT group had fewer patients with benign tumors (52.1% vs. 88.5%) and more borderline malignancy (47.9% vs. 11.5%) than the SA group. Among these clinical features, sex, laterality, extent of surgery, tumor size, age, diagnosis year, and tumor behavior code were all statistically significant (*p* < 0.05).

**TABLE 1:** Demographic, clinicopathological, and treatment characteristics of patients with non‐malignant meningioma (*n* = 8629).

Characteristic	Level	Overall	Surgery alone	Radiation after surgery	Standardize diff.	*p*‐Value
*n*		8629	7716	913		
Sex (%)					0.1 (0.0, 0.2)	0.004
Female		6075 (70.4)	5470 (70.9%)	605 (66.3%)		
Male		2554 (29.6)	2246 (29.1%)	308 (33.7%)		
Laterality (%)					0.3 (0.2, 0.3)	<0.001
Left		3386 (39.2)	2967 (38.5%)	419 (45.9%)		
Right		3373 (39.1)	3000 (38.9%)	373 (40.9%)		
Others		1870 (21.7)	1749 (22.7%)	121 (13.3%)		
OS_status (%)					0.1 (0.0, 0.1)	0.051
Alive		8192 (94.9)	7313 (94.8%)	879 (96.3%)		
Dead		437 (5.1)	403 (5.2%)	34 (3.7%)		
Surgery (%)					0.4 (0.4, 0.5)	<0.001
STR		3032 (35.1)	2544 (33.0%)	488 (53.5%)		
GTR		5597 (64.9)	5172 (67.0%)	425 (46.5%)		
Marital_status (%)					0.1 (0.0, 0.1)	0.141
Married		5012 (58.1)	4478 (58.0%)	534 (58.5%)		
Separate		1497 (17.3)	1358 (17.6%)	139 (15.2%)		
Others		2120 (24.6)	1880 (24.4%)	240 (26.3%)		
Tumor_size (%)					0.4 (0.3, 0.4)	<0.001
≤42 mm		3592 (41.6)	4782 (62.0%)	396 (43.4%)		
>42 mm		5037 (58.4)	2934 (38.0%)	517 (56.6%)		
Race (%)					0.1 (0.0, 0.1)	0.060
Black		974 (11.3)	873 (11.3%)	101 (11.1%)		
White		6537 (75.8)	5866 (76.0%)	671 (73.5%)		
Others/unknown		1118 (13.0)	977 (12.7%)	141 (15.4%)		
Year_of_diagnosis (%)					0.1 (0.1, 0.2)	0.006
2016		2061 (23.9)	1805 (23.4%)	256 (28.0%)		
2017		2314 (26.8)	2102 (27.2%)	212 (23.2%)		
2018		2206 (25.6)	1978 (25.6%)	228 (25.0%)		
2019		2048 (23.7)	1831 (23.7%)	217 (23.8%)		
Age (%)					0.2 (0.2, 0.3)	<0.001
20–39 years		828 (9.6)	721 (9.3%)	107 (11.7%)		
40–59 years		3489 (40.4)	3076 (39.9%)	413 (45.2%)		
60–79 years		3876 (44.9)	3499 (45.3%)	377 (41.3%)		
80+ years		436 (5.1)	420 (5.4%)	16 (1.8%)		
Behavior_code (%)					0.9 (0.8, 0.9)	<0.001
Benign		7302 (84.6)	6826 (88.5%)	476 (52.1%)		
Borderline malignancy		1327 (15.4)	890 (11.5%)	437 (47.9%)		
OS_time (mean ± SD)			21.5 ± 13.6	22.7 ± 13.5	0.1 (0.0, 0.2)	0.006

Abbreviations: GTR, gross total resection; OS_status, overall survival status; OS_time, overall survival time; PORT, postoperative radiotherapy; SD, standard deviation; STR, subtotal resection.

**TABLE 2:** Factors associated with PORT and surgical treatment in non‐malignant meningioma.

Dependent: Surg_Rad_Seq		Surgery alone (*N* = 7716)	PORT (*N* = 913)	OR (univariable)	OR (multivariable)
Sex					
Female		5470 (70.9%)	605 (66.3%)	Ref	
Male		2246 (29.1%)	308 (33.7%)	1.24 (1.07–1.43, *p* = 0.004)	
Laterality					
Left		2967 (38.5%)	419 (45.9%)	Ref	Ref
Right		3000 (38.9%)	373 (40.9%)	0.88 (0.76–1.02, *p* = 0.093)	0.87 (0.74–1.03, *p* = 0.103)
Others		1749 (22.7%)	121 (13.3%)	0.49 (0.40–0.60, *p* < 0.001)	0.48 (0.38–0.60, *p* < 0.001)
Surgery					
STR		2544 (33%)	488 (53.5%)	Ref	Ref
GTR		5172 (67%)	425 (46.5%)	0.43 (0.37–0.49, *p* < 0.001)	0.30 (0.26–0.35, *p* < 0.001)
Marital_status					
Married		4478 (58%)	534 (58.5%)	Ref	
Separate		1358 (17.6%)	139 (15.2%)	0.86 (0.71–1.04, *p* = 0.127)	
Others		1880 (24.4%)	240 (26.3%)	1.07 (0.91–1.26, *p* = 0.408)	
Tumor_size					
≥42 mm		3058 (39.6%)	534 (58.5%)	Ref	Ref
<42 mm		4658 (60.4%)	379 (41.5%)	0.47 (0.41–0.54, *p* < 0.001)	0.67 (0.57–0.78, *p* < 0.001)
Race					
Black		873 (11.3%)	101 (11.1%)	Ref	Ref
White		5866 (76%)	671 (73.5%)	0.99 (0.79–1.23, *p* = 0.920)	1.20 (0.94–1.53, *p* = 0.140)
Others/unknown		977 (12.7%)	141 (15.4%)	1.25 (0.95–1.64, *p* = 0.110)	1.42 (1.05–1.91, *p* = 0.021)
Year_of_diagnosis					
2016		1805 (23.4%)	256 (28%)	Ref	Ref
2017		2102 (27.2%)	212 (23.2%)	0.71 (0.59–0.86, *p* < 0.001)	0.65 (0.53–0.80, *p* < 0.001)
2018		1978 (25.6%)	228 (25%)	0.81 (0.67–0.98, *p* = 0.032)	0.71 (0.57–0.87, *p* = 0.001)
2019		1831 (23.7%)	217 (23.8%)	0.84 (0.69–1.01, *p* = 0.067)	0.77 (0.62–0.95, *p* = 0.013)
Age					
20–39 years		721 (9.3%)	107 (11.7%)	Ref	Ref
40–59 years		3076 (39.9%)	413 (45.2%)	0.90 (0.72–1.14, *p* = 0.389)	1.10 (0.85–1.41, *p* = 0.469)
60–79 years		3499 (45.3%)	377 (41.3%)	0.73 (0.58–0.91, *p* = 0.006)	0.82 (0.64–1.05, *p* = 0.121)
80+ years		420 (5.4%)	16 (1.8%)	0.26 (0.15–0.44, *p* < 0.001)	0.25 (0.14–0.44, *p* < 0.001)
Behavior_code					
Benign		6826 (88.5%)	476 (52.1%)	Ref	Ref
Borderline malignancy		890 (11.5%)	437 (47.9%)	7.04 (6.08–8.16, *p* < 0.001)	7.41 (6.30–8.72, *p* < 0.001)

Abbreviations: GTR, gross total resection; OR, odds ratio; PORT, postoperative radiotherapy; STR, subtotal resection.

### 3.2 Clinical characteristics of NMM associated with PORT

In univariate logistic regression analysis, sex, laterality, surgery, tumor size, year of diagnosis, age, and behavior code were related to PORT (Table 2). In the multivariate logistic stepwise regression analysis, except for sex, all other variables were associated with PORT; laterality (adjusted odds ratio [aOR] = 0.48, 95% Cl = 0.38–0.60; *p* < 0.001), surgery (aOR = 0.30, 95% Cl = 0.26–0.35; *p* < 0.001), tumor size (aOR = 0.67, 95% Cl = 0.57–0.78; *p* < 0.001), year of diagnosis (aOR = 0.30, 95% Cl = 0.26–0.35; *p* < 0.001), age (aOR = 0.25, 95% Cl = 0.14–0.44; *p* < 0.001), and behavior code (aOR = 7.41, 95% Cl = 6.30–8.72; *p* < 0.001) were most closely related to PORT.

Next, we assessed whether the relationship between borderline malignancy behavior and PORT was affected by other variables. Furthermore, we examined the relationship between borderline malignancy behavior and other variables (Table S1). There were strong associations between borderline malignancy behavior and sex, tumor size, race, and age (all *p* < 0.001). Borderline malignancy behavior was more common in males than that in females (male; aOR = 1.62; 95% CI = 1.43–1.84). Borderline malignancy behavior was lower in laterality (others; aOR = 0.64; 95% CI = 0.53–0.77) than that on the right side (aOR = 0.98; 95% CI = 0.86–1.12). Borderline malignancy behavior was less common in smaller tumors (<42 mm; aOR = 0.36; 95% CI = 0.32–0.41), and proportionately less common in White patients compared to that in Black patients (White; aOR = 0.68; 95% CI = 0.57–0.81). Age was also influential, with borderline malignancy behavior being proportionately less common at ages 40–59 (aOR = 0.62; 95% CI = 0.51–0.75) and 60–79 (aOR = 0.67; 95% CI = 0.55–0.82).

### 3.3 Survival disparity between PORT and SA

**FIGURE 2:**
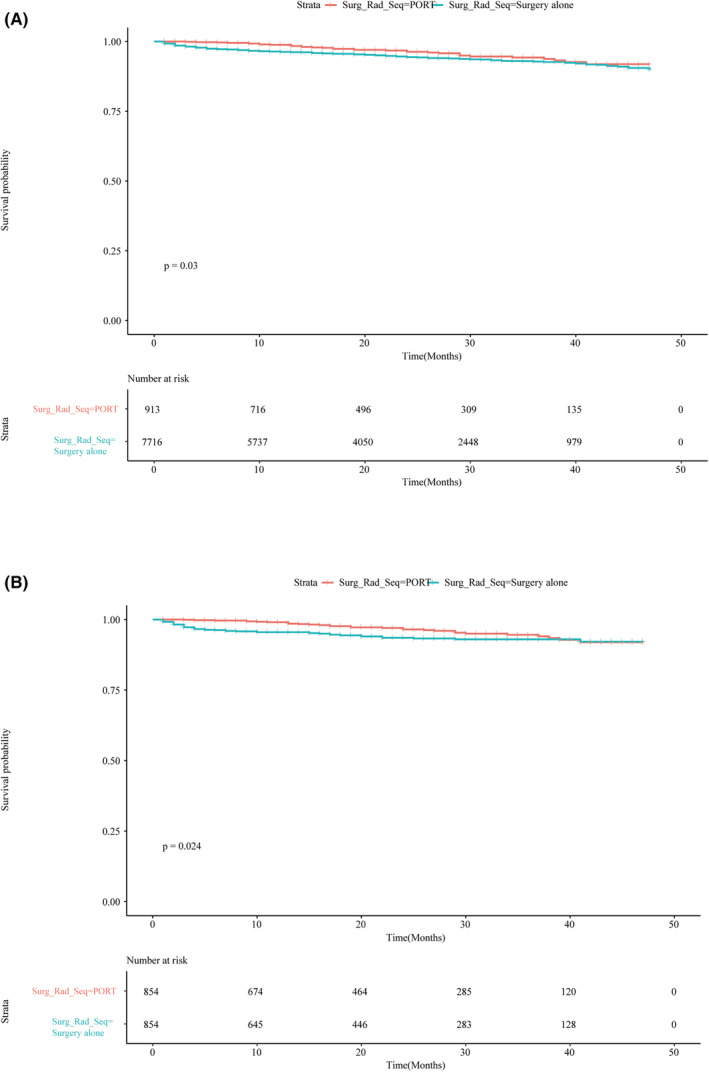
Kaplan–Meier analysis of PORT and SA groups before and after PSM. (A) Survival probability of PORT and SA treatment groups before PSM. (B) Survival probability of PORT and SA treatment groups after PSM. PORT, postoperative radiotherapy; SA, surgery alone.

The 3‐year OS rates of the SA (*n* = 7716) and PORT groups (*n* = 913) were 92.8% (95% CI 92.0%–93.6%) and 93.8% (95% CI 91.5%–96.1%) (*p* = 0.03), respectively (Figure 2A). In the univariate Cox regression analysis (Table 3), the PORT group was associated with a 32% lower risk of death than the SA group. Then, after adjusting for demographics, clinical characteristics, and treatment modality, there remained a 45% reduction in death risk in the PORT group compared to that in the SA group. Other variables, including male sex, separation, and other marital status, tumor size ≥42 mm, Black race, advanced age, and borderline malignancy, were all strongly associated with poor OS. To avoid selection bias and confounding variables, PSM was used to balance differences in clinical characteristics between the SA and PORT groups. Figure S1 shows that the distributions for the treatment and control groups were more similar than the raw data by PSM. As shown in Table S2, all variables were not statistically significant when compared before and after PSM (*p* > 0.05). Univariate Cox regression analysis showed a 40% reduction in death risk in the PORT group compared to that in the SA group. Moreover, multivariate Cox regression indicated a similar death risk (44%) in the SA (*n* = 854) and PORT groups (*n* = 854) after PSM (Table S3). The 3‐year survival rates of the SA and PORT groups were 93.0% (95% CI 91.0%–95.0%) and 94.0% (95% CI 92.4%–96.8%) (*p* = 0.024), respectively (Figure 2B). Borderline malignancy, advanced age, and separation marital status increased mortality risk both before and after PSM. Moreover, tumor size (<42 mm) and PORT also decreased mortality risk.

**TABLE 3:** Univariate and multivariate Cox regression analyses with non‐malignant meningioma clinical variables (before PSM).

Characteristics	Univariate analysis			Multivariable analysis		
	Hazard ratio	95% CI	*p*‐Value	Hazard ratio	95% CI	*p*‐Value
Sex						
Female	Ref			Ref		
Male	2.000	1.66–2.42	0.000	1.920	1.58–2.33	0.000
Laterality						
Left	Ref			Ref		
Right	0.970	0.78–1.2	0.782	0.990	0.79–1.23	0.903
Others	1.230	0.96–1.56	0.097	1.290	1.01–1.67	0.045
Surgery						
STR	Ref			Ref		
GTR	0.870	0.72–1.06	0.160	0.860	0.7–1.05	0.134
Marital_status						
Married	Ref			Ref		
Separate	2.120	1.69–2.66	0.000	1.950	1.54–2.47	0.000
Others	1.430	1.14–1.8	0.002	1.740	1.37–2.2	0.000
Tumor_size						
≥42	Ref			Ref		
<42	0.570	0.47–0.69	0.000	0.620	0.51–0.76	0.000
Race						
Black	Ref			Ref		
White	0.660	0.51–0.86	0.002	0.640	0.5–0.84	0.001
Others/unknown	0.520	0.35–0.76	0.001	0.520	0.35–0.77	0.001
Year_of_diagnosis						
2016	Ref			Ref		
2017	0.960	0.75–1.22	0.751	0.950	0.74–1.21	0.659
2018	1.020	0.77–1.34	0.904	0.960	0.73–1.27	0.782
2019	1.120	0.8–1.58	0.507	1.100	0.78–1.55	0.587
Age						
20–39 years	Ref			Ref		
40–59 years	0.980	0.58–1.64	0.929	1.130	0.67–1.89	0.654
60–79 years	3.370	2.09–5.43	0.000	3.750	2.31–6.09	0.000
80+ years	8.410	5.02–14.09	0.000	8.140	4.8–13.78	0.000
Behavior_code						
Benign	Ref			Ref		
Borderline malignancy	1.810	1.46–2.26	0.000	1.720	1.35–2.18	0.000
Surg_Rad_Seq						
Surgery alone	Ref			Ref		
PORT	0.680	0.48–0.96	0.030	0.550	0.38–0.8	0.002

Abbreviations: 95% CI, 95% confidence interval; GTR, gross total resection; PORT, postoperative radiotherapy; STR, subtotal resection.

### 3.4 NMM clinical subgroup analysis according to PORT

**FIGURE 3:**
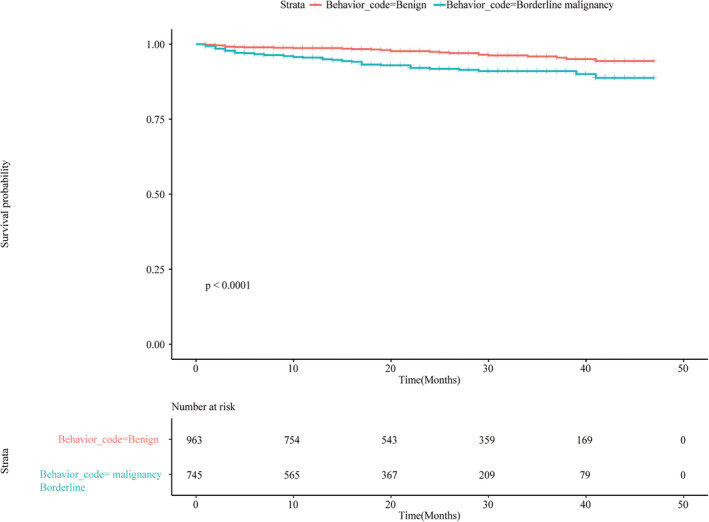
Kaplan–Meier analysis of benign and borderline malignancy behavior in PORT group after propensity score matching.

We performed a subgroup analysis of patients who underwent PORT to determine which patients with NMM showed OS benefit from PORT management. The results showed that OS for borderline malignant tumors (HR 1.23, 95% CI 1.07–1.41, *p* = 0.004) did not benefit from PORT treatment, whereas the OS for right‐sided tumors (HR 0.87, 95% CI 0.75–1.01, *p* = 0.060) did improve (Table 4). The KM survival analysis showed that the 3‐year survival rate for tumors showing borderline malignant behavior (91.0%, 95% CI 0.885–0.936) was poorer than that for tumors showing benign behavior (95.5%, 95% CI 0.936–0.974) (*p* < 0.0001) (Figure 3).

**TABLE 4:** Subgroup analysis of PORT group by multivariate Cox regression.

Characteristics	Univariate analysis		
	Hazard ratio	95% CI	*p*‐Value
Sex			
Female			
Male	0.99	0.85–1.14	0.845
Laterality			
Left			
Right	0.87	0.75–1.01	0.060
Others	0.88	0.71–1.08	0.222
Surgery			
STR			
GTR	1.01	0.88–1.15	0.937
Marital_status			
Married			
Separate	0.86	0.70–1.05	0.128
Others	1.03	0.88–1.21	0.727
Tumor_size			
≥42 mm			
<42 mm	0.94	0.82–1.08	0.357
Race			
Black			
White	0.89	0.71–1.11	0.289
Others/unknown	0.80	0.61–1.05	0.102
Age			
20–39 years			
40–59 years	1.01	0.81–1.26	0.925
60–79 years	1.09	0.87–1.37	0.446
80+ years	1.16	0.67–2.00	0.593
Behavior_code			
Benign			
Borderline malignancy	1.23	1.07–1.41	0.004

Abbreviations: 95% CI, 95% confidence interval; GTR, gross total resection; PORT, postoperative radiotherapy; STR, subtotal resection.

## 4 DISCUSSION

In this study, based on a large population database analysis, we investigated differences in population characteristics, clinical data, and OS of patients with NMM treated with PORT or SA. Compared with the patients in the SA group, those in the PORT group were more likely to be female, have left sided tumors, have underwent STR, have larger tumor size (≥42 mm), be aged between 40 and 59 years, and have tumors categorized as showing benign behavior. Patients in the PORT group showed better OS than those in the SA group.

In an analysis of patients with NMM receiving PORT and SA treatment, tumors showing borderline malignancy and benign behavior were strongly associated with PORT treatment. As the incidence of tumors showing benign behavior is 7–8 times higher than that of borderline malignancy, this may partly explain the survival benefit observed in the PORT group. However, the incidence of tumors showing benign and borderline malignancy behavior treated with PORT was almost similar. Total surgical excision is the treatment of choice for the most prevalent benign meningiomas, as determined by a consensus. Radiotherapy is performed for recurrent or persistent tumor growth following surgical excision.^16^, ^17^ Although surgery is the preferred treatment option for benign tumors, tumors with benign behavior receiving PORT behaved similar to tumors showing borderline malignancy behavior. We also noticed that the association of borderline malignancy behavior with PORT was perturbed by other demographic and clinical characteristics of NMM. The characteristics of patients with tumors showing benign or borderline malignancy behavior receiving PORT await further confirmation.

Our results showed that a higher proportion of women who received PORT treatment than those who received SA. Meningiomas are significantly more common in women than those in men.^2^, ^18^ The mechanism may be related to differences in sex hormone expression and receptors. Administration of exogenous sex hormone therapy or progesterone analysis could reduce meningioma risk.^19^, ^20^ Whether PORT affected hormone expression was unclear. Left‐sided meningiomas were highly associated with aphasia or mental disorders, and tumor resection significantly changed the patient's language function, which may be a factor that might have affected clinicians' plans. However, the tumor laterality is unimportant in the patient's quality of life.^21^, ^22^ This was consistent with our study; laterality of tumors in those receiving PORT did not seem to affect the survival rate but only changed the patient's intuitive feeling. PORT significantly reduced tumor size, although when GTR or STR should be used is still debated. For benign meningioma, clinicians (90%) do not recommend PORT, and PORT was abandoned after GTR for Grade II meningioma, while it was highly preferred after STR.^23^, ^24^, ^25^ There is still lack of evidence to explain these results.

After PSM, patient characteristics were eliminated to the greatest extent possible. The 3‐year survival rate of patients with NMM was similar between the SA and PORT groups (both before and after PSM). Furthermore, compared to the SA group, the PORT group had a greater reduction in death risk in univariate and multivariate Cox regression analyses. In the PORT subgroup analysis, multivariate Cox regression showed similar results to logistic regression, with borderline malignancy behavior comparable to benign behavior with a 23% increased death risk after PORT treatment (*p* = 0.004). However, laterality, tumor size, race, and age did not appear to influence survival benefit in those receiving PORT (*p* > 0.05).

Treatment of NMM with borderline malignant behavior is currently controversial. Different organizations have different views on how to treat atypical meningiomas, with some preferring treatment with radiation therapy after GTR, while others preferring radiation therapy alone.^23^ NCCN and EANO guidelines recommend PORT for incompletely resected Grade II tumors.^8^, ^26^ The randomized trial ROAM/EORTC demonstrated a clinical benefit of PORT for recurrent atypical meningiomas after complete surgery.^27^ Prospective clinical trials are still needed to overcome this uncertainty.

To the best of our knowledge, this is the first study to report analysis of different clinical features of NMM receiving PORT. We believe that our study, which included PSM to exclude relevant confounding factors, suggests the potential applications of PORT in different populations. PORT showed a promising therapeutic advantage over SA and was more suitable for patients with tumors showing borderline malignant behavior. However, it did not perform well for different surgical treatment modalities (GTR and STR) or tumor sizes (≥42 mm and <42 mm).

Our research has several limitations. First, the SEER database does not include patient‐specific clinical information, including gene expression‐related data. Existing studies have confirmed that inactivation of the most common NF2 tumor suppressor gene^28^ and TRAF7, AKT1, and KLF4 mutations affect meningiomas.^29^, ^30^, ^31^ Second, the non‐randomized distribution of patients receiving radiotherapy in the SEER dataset may have influenced the apparent clinical efficacy of radiotherapy.^32^ Our study applied the PSM method to control potential bias as much as possible.^33^ Finally, the specific methods of surgery, different surgical approaches, and surgical residues will all contribute to the therapeutic effect of PORT. Due to the long survival of patients with NMM, conventional survival analysis may be weak, and the risks for tumor progression are still worthy of consideration. However, there is a lack of recurrence data and a small amount of tumor‐specific death data in the SEER database itself, making such analysis difficult. We look forward to continuing to collect PORT meningioma data in the future to extend the findings of this study.

## 5 CONCLUSIONS

This was a large‐scale population‐based study that comprehensively delineated the differences in demographics, clinical characteristics, and prognosis between patients receiving PORT and surgical meningioma resection. PORT had a better prognostic benefit than SA, and after adjustment for other covariates by PSM, only tumors exhibiting benign behaviors were found to be benefited from PORT treatment. We recommend the aggressive implementation of PORT in patients with benign meningioma. More research is required to determine specific treatment options for PORT in NMM.

## AUTHOR CONTRIBUTIONS

**Yongan Jiang:** Data curation (equal); methodology (equal); writing – original draft (equal). **Peng Chen:** Data curation (equal); methodology (equal); writing – review and editing (equal). **JiaWei Liang:** Data curation (equal); methodology (equal); writing – review and editing (equal). **JiaHong Cai:** Formal analysis (equal). **Yi Zhang:** Formal analysis (equal). **HengYi Fan:** Writing – original draft (equal). **RaoRao Yuan:** Investigation (equal); validation (equal). **WenXing Zheng:** Software (equal); validation (equal). **ShiQi Cheng:** Conceptualization (equal); funding acquisition (equal). **Yan Zhang:** Conceptualization (equal); funding acquisition (equal); project administration (equal).

## FUNDING INFORMATION

JiangXi Provincial Health Commission Science and Technology Program (No. 202130385). JiangXi Provincial Education Bureau Program (No. GJJ210118). National Natural Science Foundation of China (No. 82260388). Jiangxi Administration of Traditional Chinese Medicine Program (No. 2022Z017).

## CONFLICT OF INTEREST STATEMENT

This research does not include any research conducted by any author on human participants or animals. The authors declare no competing interests.

## Supporting information


Figure S1:

Table S1:

Table S2:

Table S3:

## Data Availability

The data of this study were downloaded and compiled from National Cancer Institute's Surveillance, Epidemiology and End Results (SEER) database (https://seer.cancer.gov/). The data used to support the results of this study were obtained from the corresponding author.
