# Supplementary figures and images for: Surveillance, Epidemiology, and End Results database and propensity score matching analysis of postoperative radiotherapy for non‐malignant meningioma: A retrospective cohort study
Source: Cancer Med. 2023 May 31;12(14):15054–64. doi: 10.1002/cam4.6177 (PMC10417067; doi:10.1002/cam4.6177)

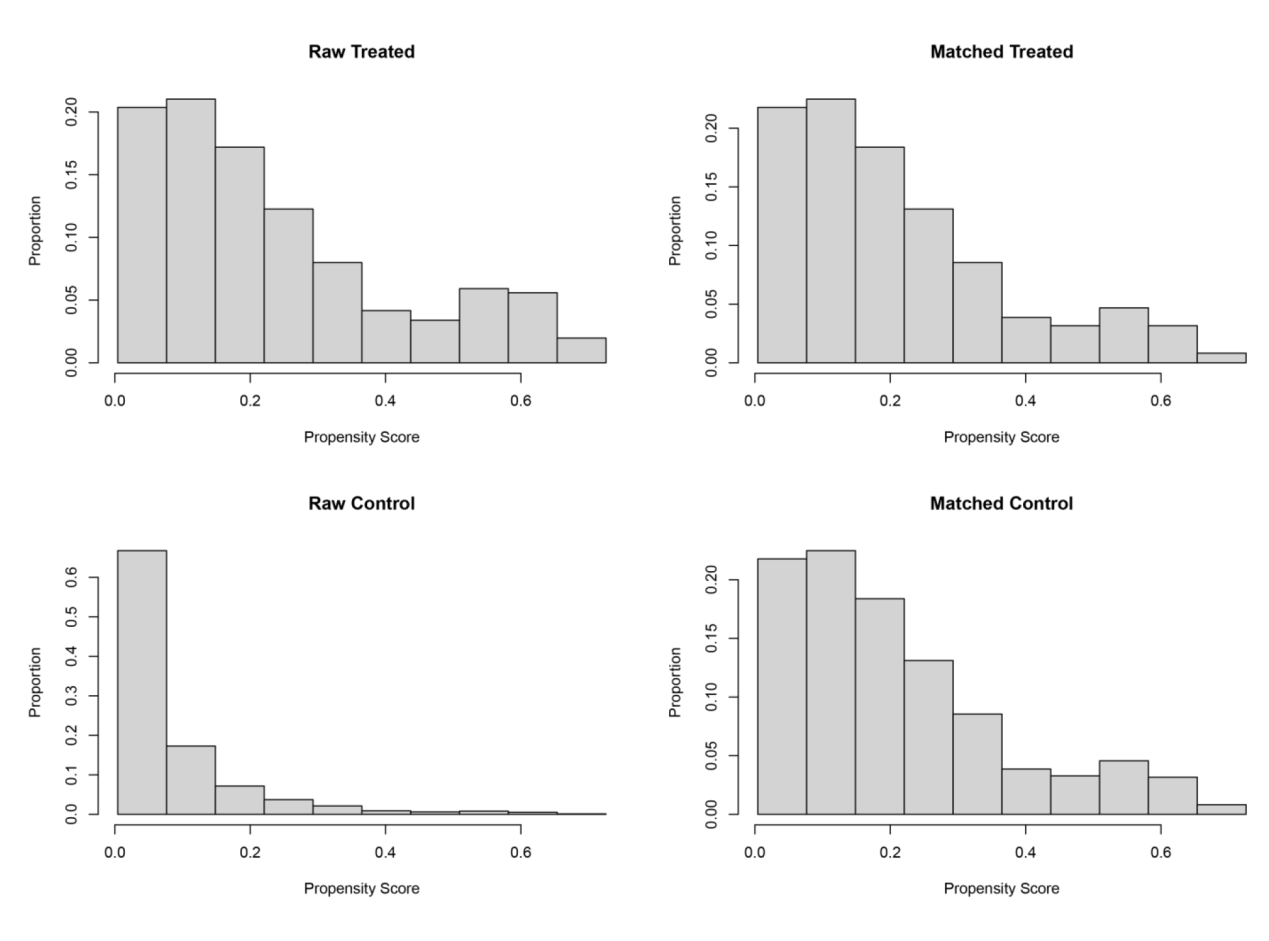


**Figure S1:** Propensity score matching was applied to the assessment data (1:1, control vs. treated).

Supplement: Supplementary file 1 — Figure S1: [file CAM4-12-15054-s001.docx]
