# Supplementary material for: Surveillance, Epidemiology, and End Results database and propensity score matching analysis of postoperative radiotherapy for non‐malignant meningioma: A retrospective cohort study
Source: Cancer Med. 2023 May 31;12(14):15054–64. doi: 10.1002/cam4.6177 (PMC10417067; doi:10.1002/cam4.6177)
Supplement: Supplementary file 2 — Table S1: [file CAM4-12-15054-s003.docx]

**Table S1:** Interaction between borderline malignancy and other factors associated with PORT.

| **Dependent: Behavior_code** |  | **Benign (N=7302)** | **Borderline malignancy (N=1327)** | **OR (univariable)** | **OR (multivariable)** | **OR (final)** |
| --- | --- | --- | --- | --- | --- | --- |
| **Sex** | **Female** | 5299 (72.6%) | 776 (58.5%) |  |  |  |
|  | **Male** | 2003 (27.4%) | 551 (41.5%) | 1.88 (1.66-2.12, *p*<0.001) | 1.62 (1.43-1.84, *p*<0.001) | 1.62 (1.43-1.84, *p*<0.001) |
| **Laterality** | **Left** | 2806 (38.4%) | 580 (43.7%) |  |  |  |
|  | **Right** | 2805 (38.4%) | 568 (42.8%) | 0.98 (0.86-1.11, *p*=0.751) | 0.98 (0.86-1.12, *p*=0.800) | 0.98 (0.86-1.12, *p*=0.798) |
|  | **Others** | 1691 (23.2%) | 179 (13.5%) | 0.51 (0.43-0.61, *p*<0.001) | 0.64 (0.53-0.77, *p*<0.001) | 0.64 (0.53-0.77, *p*<0.001) |
| **Surgery** | **STR** | 2631 (36%) | 401 (30.2%) |  |  |  |
|  | **GTR** | 4671 (64%) | 926 (69.8%) | 1.30 (1.15-1.48, *p*<0.001) | 1.15 (1.01-1.31, *p*=0.038) | 1.15 (1.01-1.31, *p*=0.038) |
| **Marital_status** | **Married** | 4249 (58.2%) | 763 (57.5%) |  |  |  |
|  | **Separate** | 1290 (17.7%) | 207 (15.6%) | 0.89 (0.76-1.05, *p*=0.183) | 0.97 (0.82-1.16, *p*=0.748) |  |
|  | **Others** | 1763 (24.1%) | 357 (26.9%) | 1.13 (0.98-1.29, *p*=0.087) | 1.04 (0.89-1.20, *p*=0.640) |  |
| **Tumor_size** | **≥42mm** | 2734 (37.4%) | 858 (64.7%) |  |  |  |
|  | **<42mm** | 4568 (62.6%) | 469 (35.3%) | 0.33 (0.29-0.37, *p*<0.001) | 0.36 (0.32-0.41, *p*<0.001) | 0.36 (0.32-0.41, *p*<0.001) |
| **Race** | **Black** | 779 (10.7%) | 195 (14.7%) |  |  |  |
|  | **White** | 5578 (76.4%) | 959 (72.3%) | 0.69 (0.58-0.82, *p*<0.001) | 0.69 (0.57-0.82, *p*<0.001) | 0.68 (0.57-0.81, *p*<0.001) |
|  | **Others/Unknown** | 945 (12.9%) | 173 (13%) | 0.73 (0.58-0.92, *p*=0.007) | 0.71 (0.56-0.89, *p*=0.004) | 0.70 (0.55-0.88, *p*=0.003) |
| **Year_of_diagnosis** | **2016** | 1778 (24.3%) | 283 (21.3%) |  |  |  |
|  | **2017** | 1962 (26.9%) | 352 (26.5%) | 1.13 (0.95-1.33, *p*=0.165) | 1.12 (0.94-1.33, *p*=0.215) | 1.12 (0.94-1.33, *p*=0.213) |
|  | **2018** | 1840 (25.2%) | 366 (27.6%) | 1.25 (1.06-1.48, *p*=0.009) | 1.25 (1.05-1.49, *p*=0.011) | 1.25 (1.05-1.49, *p*=0.011) |
|  | **2019** | 1722 (23.6%) | 326 (24.6%) | 1.19 (1.00-1.41, *p*=0.049) | 1.21 (1.01-1.44, *p*=0.036) | 1.21 (1.01-1.44, *p*=0.037) |
| **Age** | **20-39 years** | 653 (8.9%) | 175 (13.2%) |  |  |  |
|  | **40-59 years** | 2997 (41%) | 492 (37.1%) | 0.61 (0.51-0.74, *p*<0.001) | 0.62 (0.51-0.76, *p*<0.001) | 0.62 (0.51-0.75, *p*<0.001) |
|  | **60-79 years** | 3282 (44.9%) | 594 (44.8%) | 0.68 (0.56-0.82, *p*<0.001) | 0.68 (0.56-0.83, *p*<0.001) | 0.67 (0.55-0.82, *p*<0.001) |
|  | **80+ years** | 370 (5.1%) | 66 (5%) | 0.67 (0.49-0.91, *p*=0.010) | 0.67 (0.49-0.93, *p*=0.018) | 0.66 (0.48-0.91, *p*=0.011) |

PORT, postoperative radiotherapy; OR, odds ratio; GTR, gross total resection; STR, subtotal resection.
