# Supplementary material for: Surveillance, Epidemiology, and End Results database and propensity score matching analysis of postoperative radiotherapy for non‐malignant meningioma: A retrospective cohort study
Source: Cancer Med. 2023 May 31;12(14):15054–64. doi: 10.1002/cam4.6177 (PMC10417067; doi:10.1002/cam4.6177)
Supplement: Supplementary file 3 — Table S2: [file CAM4-12-15054-s002.docx]

**Table S2:** Factors before and after propensity score matching.

|  |  | **pre_PSM** | | | **PSM** | | |
| --- | --- | --- | --- | --- | --- | --- | --- |
| **Characteristic** | **Overall** | **Surgery** | **Postoperative Radiotherapy** | ***p*.value** | **Surgery** | **Postoperative Radiotherapy** | ***p*.value** |
|  | N=8629 | N=7716 | N=913 |  | N=854 | N=854 |  |
| **Sex (%)** |  |  |  |  |  |  |  |
| **Female** | 6075 (70.4) | 5470 (70.9) | 605 (66.3) | 0.004 | 563 (65.9) | 573 (67.1) | 0.644 |
| **Male** | 2554 (29.6) | 2246 (29.1) | 308 (33.7) |  | 291 (34.1) | 281 (32.9) |  |
| **Laterality (%)** |  |  |  |  |  |  |  |
| **Left** | 3386 (39.2) | 2967 (38.5) | 419 (45.9) | <0.001 | 394 (46.1) | 385 (45.1) | 0.600 |
| **Right** | 3373 (39.1) | 3000 (38.9) | 373 (40.9) |  | 356 (41.7) | 351 (41.1) |  |
| **Others** | 1870 (21.7) | 1749 (22.7) | 121 (13.3) |  | 104 (12.2) | 118 (13.8) |  |
| **Surgery (%)** |  |  |  |  |  |  |  |
| **STR** | 3032 (35.1) | 2544 (33.0) | 488 (53.5) | <0.001 | 436 (51.1) | 432 (50.6) | 0.885 |
| **GTR** | 5597 (64.9) | 5172 (67.0) | 425 (46.5) |  | 418 (48.9) | 422 (49.4) |  |
| **Marital_status (%)** |  |  |  |  |  |  |  |
| **Married** | 5012 (58.1) | 4478 (58.0) | 534 (58.5) | 0.141 | 519 (60.8) | 499 (58.4) | 0.592 |
| **Separate** | 1497 (17.3) | 1358 (17.6) | 139 (15.2) |  | 128 (15.0) | 132 (15.5) |  |
| **Others** | 2120 (24.6) | 1880 (24.4) | 240 (26.3) |  | 207 (24.2) | 223 (26.1) |  |
| **Tumor_size (%)** |  |  |  |  |  |  |  |
| **≥42mm** | 3592 (41.6) | 3058 (39.6) | 534 (58.5) | <0.001 | 499 (58.4) | 485 (56.8) | 0.524 |
| **<42mm** | 5037 (58.4) | 4658 (60.4) | 379 (41.5) |  | 355 (41.6) | 369 (43.2) |  |
| **Race (%)** |  |  |  |  |  |  |  |
| **Black** | 974 (11.3) | 873 (11.3) | 101 (11.1) | 0.060 | 88 (10.3) | 93 (10.9) | 0.662 |
| **White** | 6537 (75.8) | 5866 (76.0) | 671 (73.5) |  | 647 (75.8) | 631 (73.9) |  |
| **Others/Unknown** | 1118 (13.0) | 977 (12.7) | 141 (15.4) |  | 119 (13.9) | 130 (15.2) |  |
| **Age (%)** |  |  |  |  |  |  |  |
| **20-39 years** | 828 ( 9.6) | 721 ( 9.3) | 107 (11.7) | <0.001 | 94 (11.0) | 98 (11.5) | 0.442 |
| **40-59 years** | 3489 (40.4) | 3076 (39.9) | 413 (45.2) |  | 371 (43.4) | 380 (44.5) |  |
| **60-79 years** | 3876 (44.9) | 3499 (45.3) | 377 (41.3) |  | 380 (44.5) | 360 (42.2) |  |
| **80+ years** | 436 ( 5.1) | 420 ( 5.4) | 16 ( 1.8) |  | 9 ( 1.1) | 16 ( 1.9) |  |
| **Year_of_diagnosis (%)** |  |  |  |  |  |  |  |
| **2016** | 2061 (23.9) | 1805 (23.4) | 256 (28.0) | 0.006 | 229 (26.8) | 231 (27.0) | 0.933 |
| **2017** | 2314 (26.8) | 2102 (27.2) | 212 (23.2) |  | 210 (24.6) | 205 (24.0) |  |
| **2018** | 2206 (25.6) | 1978 (25.6) | 228 (25.0) |  | 224 (26.2) | 217 (25.4) |  |
| **2019** | 2048 (23.7) | 1831 (23.7) | 217 (23.8) |  | 191 (22.4) | 201 (23.5) |  |
| **Behavior_code (%)** |  |  |  |  |  |  |  |
| **Benign** | 7302 (84.6) | 6826 (88.5) | 476 (52.1) | <0.001 | 487 (57.0) | 476 (55.7) | 0.626 |
| **Borderline malignancy** | 1327 (15.4) | 890 (11.5) | 437 (47.9) |  | 367 (43.0) | 378 (44.3) |  |

GTR, gross total resection; STR, subtotal resection; PSM, propensity score matching.
