# Supplementary material for: Surveillance, Epidemiology, and End Results database and propensity score matching analysis of postoperative radiotherapy for non‐malignant meningioma: A retrospective cohort study
Source: Cancer Med. 2023 May 31;12(14):15054–64. doi: 10.1002/cam4.6177 (PMC10417067; doi:10.1002/cam4.6177)
Supplement: Supplementary file 4 — Table S3: [file CAM4-12-15054-s004.docx]

**Table S3:** Results of univariate and multivariate Cox regression analyses after PSM.

|  | **Univariate analysis** | | | **Multivariable analysis** | | |
| --- | --- | --- | --- | --- | --- | --- |
| **Characteristics** | **Hazard.Ratio** | **95% CI** | ***p*.value** | **Hazard.Ratio** | **95% CI** | ***p*.value** |
| **Sex** |  |  |  |  |  |  |
| **Female** | Ref | | | Ref | | |
| **Male** | 1.42 | 0.91-2.22 | 0.126 | 1.21 | 0.77-1.92 | 0.409 |
| **Laterality** |  |  |  |  |  |  |
| **Left** | Ref | | | Ref | | |
| **Right** | 0.76 | 0.46-1.24 | 0.272 | 0.76 | 0.46-1.26 | 0.289 |
| **Others** | 1.26 | 0.68-2.32 | 0.465 | 1.44 | 0.77-2.7 | 0.258 |
| **Surgery** |  |  |  |  |  |  |
| **STR** | Ref | | | Ref | | |
| **GTR** | 0.96 | 0.62-1.5 | 0.874 | 0.81 | 0.51-1.28 | 0.364 |
| **Marital_status** |  |  |  |  |  |  |
| **Married** | Ref | | | Ref | | |
| **Separate** | 2.69 | 1.57-4.61 | 0.000 | 2.57 | 1.46-4.52 | 0.001 |
| **Others** | 1.77 | 1.04-2.99 | 0.034 | 2.07 | 1.21-3.56 | 0.008 |
| **Tumor_size** |  |  |  |  |  |  |
| **≥42** | Ref | | | Ref | | |
| **<42** | 0.37 | 0.22-0.63 | 0.000 | 0.46 | 0.27-0.8 | 0.006 |
| **Race** |  |  |  |  |  |  |
| **Black** | Ref | | | Ref | | |
| **White** | 0.57 | 0.3-1.05 | 0.070 | 0.61 | 0.33-1.15 | 0.127 |
| **Others/Unknown** | 0.58 | 0.26-1.29 | 0.180 | 0.6 | 0.26-1.39 | 0.236 |
| **Year_of_diagnosis** |  |  |  |  |  |  |
| **2016** | Ref | | | Ref | | |
| **2017** | 0.95 | 0.54-1.69 | 0.870 | 0.85 | 0.48-1.52 | 0.591 |
| **2018** | 1.02 | 0.53-1.96 | 0.945 | 0.78 | 0.41-1.52 | 0.470 |
| **2019** | 1.40 | 0.63-3.08 | 0.408 | 1.17 | 0.53-2.59 | 0.700 |
| **Age** |  |  |  |  |  |  |
| **20-39 years** | Ref | | | Ref | | |
| **40-59 years** | 4.42 | 0.59-33.18 | 0.149 | 5.2 | 0.69-39.25 | 0.110 |
| **60-79 years** | 15.57 | 2.16-112.48 | 0.006 | 16.93 | 2.32-123.35 | 0.005 |
| **80+ years** | 39.47 | 4.41-353.27 | 0.001 | 36.82 | 3.98-340.83 | 0.001 |
| **Behavior_code** |  |  |  |  |  |  |
| **Benign** | Ref | | | Ref | | |
| **Borderline malignancy** | 2.60 | 1.64-4.13 | 0.000 | 0.56 | 0.35-0.88 | 0.013 |
| **Surg_Rad_Seq** |  |  |  |  |  |  |
| **Surgery** | Ref | | | Ref | | |
| **PORT** | 0.60 | 0.38-0.94 | 0.025 | 0.56 | 0.35-0.88 | 0.013 |

PORT, postoperative radiotherapy; GTR, gross total resection; STR, subtotal resection; 95% CI, 95% confidence interval.
